# Clinical peripherality: development of a peripherality index for rural health services

**DOI:** 10.1186/1472-6963-8-23

**Published:** 2008-01-25

**Authors:** Gillian M Swan, Sivasubramaniam Selvaraj, David J Godden

**Affiliations:** 1 Centre for Rural Health, University of Aberdeen, The Green House, Beechwood Business Park, Inverness, Scotland IV2 3BL, UK

## Abstract

**Background:**

The configuration of rural health services is influenced by geography. Rural health practitioners provide a broader range of services to smaller populations scattered over wider areas or more difficult terrain than their urban counterparts. This has implications for training and quality assurance of outcomes. This exploratory study describes the development of a "clinical peripherality" indicator that has potential application to remote and rural general practice communities for planning and research purposes.

**Methods:**

Profiles of general practice communities in Scotland were created from a variety of public data sources. Four candidate variables were chosen that described demographic and geographic characteristics of each practice: population density, number of patients on the practice list, travel time to nearest specialist led hospital and travel time to Health Board administrative headquarters. A clinical peripherality index, based on these variables, was derived using factor analysis. Relationships between the clinical peripherality index and services offered by the practices and the staff profile of the practices were explored in a series of univariate analyses.

**Results:**

Factor analysis on the four candidate variables yielded a robust one-factor solution explaining 75% variance with factor loadings ranging from 0.83 to 0.89. Rural and remote areas had higher median values and a greater scatter of clinical peripherality indices among their practices than an urban comparison area. The range of services offered and the profile of staffing of practices was associated with the peripherality index.

**Conclusion:**

Clinical peripherality is determined by the nature of the practice and its location relative to secondary care and administrative and educational facilities. It has features of both gravity model-based and travel time/accessibility indicators and has the potential to be applied to training of staff for rural and remote locations and to other aspects of health policy and planning. It may assist planners in conceptualising the effects on general practices of centralising specialist clinical services or administrative and educational facilities.

## Background

Planners of rural health services need to address the range of services provided at local and distant sites and ensure that training of health professionals is appropriate to their location and work pattern. The configuration of health services affects access for citizens, and tradeoffs of geographical factors against other measures of accessibility such as waiting times, costs and socio-economic factors are inevitable [1]. Studies from the United States show that rural residents often incur long travel times to access health care [2,3] and may choose therefore to use local generalist services rather than travel to see a specialist [2].

In Scotland, local health services in rural and remote areas have evolved in response to varied geography and to demographic, historical and societal events and trends. There is currently much debate about the most appropriate configuration of services, specifically the range of services to be offered and the skills required by healthcare workers to provide care at any particular level. For example, should family doctors (general practitioners) carry our minor surgical procedures if specialists are unavailable locally, and if so, how should they be trained? Previous studies of rural health in Scotland indicate that the pattern of services provided at remote and rural general practices differs from urban based practice, with higher consultation rates, differing nature of consultations, and a wider range of generalist services provided by individual practitioners [4]. Rural practices often serve small populations scattered over wider areas or more difficult terrain than their urban counterparts.

In discussing spatial accessibility of primary care Guagliardo [5] identifies several dimensions of spatial accessibility including provider to population ratios, travel impedance measures and gravity models, as well as aspatial dimensions such as affordability and culture. In this exploratory study, we have formulated a "clinical peripherality" indicator that takes account of the spatial accessibility factors and applied it to our general practice communities. Peripherality indicators have been widely studied in relation to economic and social characteristics of areas. In general, they fall into two main types: gravity model-based methodologies, which estimate economic or market potential; and travel time/cost or accessibility indicators [6]. In gravity model methods, both the proximity of a location to other economic centres and the economic size of these centres contribute to its peripherality. In contrast, in travel time/cost models, the peripherality of a location is defined by a function of the costs of reaching other major centres, the number of people that can be accessed at any chosen time from the location or the costs associated with reaching a chosen number of people from that location. In general, concepts of peripheral economic disadvantage contain three broad groups of elements: causal, contingent and associated. The first group comprises travel and transport costs and agglomerative disadvantage (lack of economies of scale); the second group is in part determined by the first and may include issues such as high costs of service provision, and weak influence on governance; whilst the third group may include issues such as poorly developed local infrastructure and sparsity of population [7]. Similar considerations may apply to the provision of health services.

We wished to examine if a clinical peripherality indicator could be developed that would relate to the pattern of services provided and therefore the training requirements for practitioners in rural and remote practices. The study had two Phases, a regional study in the West Highlands of Scotland, followed by a national study of all non-urban practices in Scotland.

## Methods

### Phase I study

**Figure 1:**
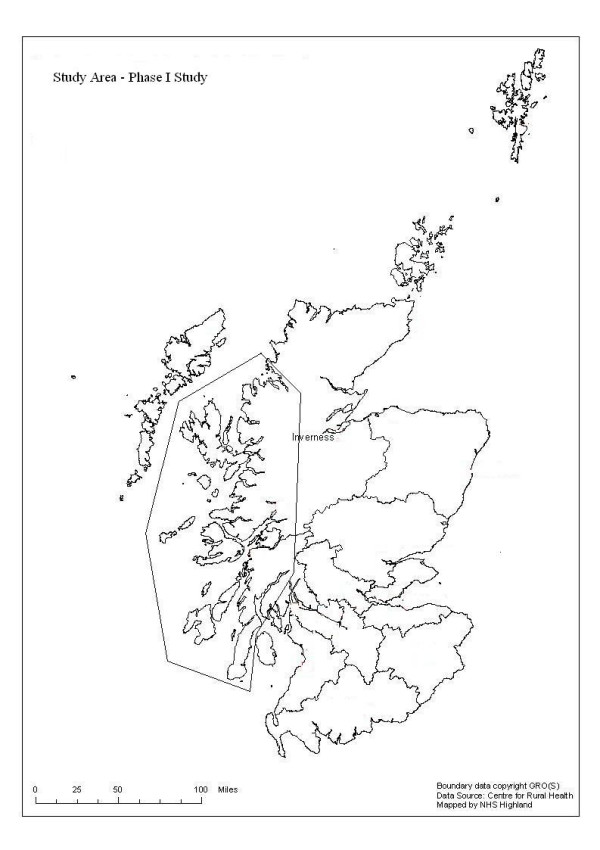
The Phase I study area.

The Phase I study was based in three rural and remote Local Health Care Cooperatives (LHCCs) in the West Highlands of Scotland: Argyll and Bute, Lochaber, and Wester Ross, Skye and Lochalsh, areas including mainland and island communities (Figure 1). A fourth LHCC in Inverness, the capital city of the Highland region, was included as an urban comparator. LHCCs were groupings of general practices assembled on a geographic basis for service provision and administrative purposes. Together the remote and rural LHCCs provided services to over 96,000 people in a geographic area of approximately 7,000 square miles, from 52 main general practice sites and 7 branch practice premises (Table 1). The practices also provided cover for 9 community hospitals. The Inverness LHCC incorporated 12 general practices, serving approximately 61,000 patients in the city and suburbs.

**Table 1:** Details of participating LHCCs

LHCC	General Practices	Branch practices	GPs (Whole time equivalent)	Patients
Argyll and Bute	30	6	66.23	64,433
Wester Ross, Skye and Lochalsh	11	1	27.75	11,363
Lochaber	11	0	23.3	20,537
Inverness	12	0	43.5	60,950

Using a variety of public and institutional data sources, a profile of each general practice and its community was prepared. For each of the 59 rural and remote practice sites, an initial detailed profile was created containing 78 items. These included geographic and demographic characteristics, deprivation scores for the practice locality, services provided by the practice such as dedicated clinics for specific conditions, a profile of practice staff, and additional roles taken on by the GP such as rescue team, police surgeon and others. The location of each practice community was matched to the appropriate Scottish Household Survey (SHS) 8-fold classification of settlements [8] using data from the Platform project [9]. The distribution of settlement types within the LHCC geographical areas is shown in Table 2.

**Table 2:** Distribution of settlement types in each study area categorised according to Scottish Household Survey Classification [8]

SHOS category number and description	West Highland	Inverness
2: Urban areas (10,000 – 125,000 population)	5%	84%
3: Accessible small towns (3 – 10,000 population within 30 minutes drive of a settlement of 10,000 or more)	5%	16%
4–7: Remote rural settlements and remote small towns (30–60 minutes from a settlement of 10,000 or more)	17%	0%
8: Very remote rural (settlements of <3,000 and over 60 minutes from a settlement of >= 10,000)	73%	0%

Population density within the ward served by each practice was derived from 2001 census data [10]. Road travel distances and times to the nearest hospital that had specialist led facilities for medical and surgical emergencies and to the area Health Board headquarters, the latter representing a locus for decision-making powers and access to education, were included as indicators of accessibility. These were calculated from the unit postcodes of the general practice premises to the unit postcode of the relevant hospital or Health Board. A variety of transport databases and internet route planners were used to derive drive times, ferry crossing durations and mileage to key NHS locations. Travel times took account of factors such as adverse terrain with poor quality roads and the necessity to use ferries in some cases. An assumption of no wait time for ferry crossings was made.

The profiles were circulated to the general practice staff for comment and correction. 49 (83%) of the profiles were returned, with corrections noted by the practices to only 18 of the 4602 data fields (0.39%). Following comments from respondents, further data were added to create a final profile containing 115 items describing each practice community.

In order to create the clinical peripherality index, four candidate variables were chosen based on previous literature. We were seeking variables that would reflect the characteristics of the practice and its location. It is known that in more remote and sparsely populated areas practices are likely to have smaller list sizes and to offer a different range of services to their urban counterparts [4]. Practice list size, i.e. the number of patients registered with the practice, and population density in the area served by the practice were therefore included as candidate variables. Travel time to the nearest specialist led hospital and Health Board headquarters completed the candidate variables. Travel time, rather than distance, has been shown to better reflect access to health services [1,3,11,12].

The Stata command Factor (Stata 9.0) was used for the analysis. Principal component method of factor extraction on the four candidate variables was employed, followed by the maximum likelihood method as an additional check. Practice list size, ward population density and travel time to hospital were log transformed to achieve near normality. The relationships among the variables were assessed by matrix plots and correlation coefficients. The peripherality index was then derived by ranking the general practices by the factor score generated from the analysis and dividing each value by the maximum rank. This was further multiplied by 100 for the index to range from 0 to 100 with a midpoint of 50. Higher values represent greater peripherality.

The relationship between the clinical peripherality index and characteristics of the 49 practices that returned the profiles were explored in a series of univariate analyses using the Wilcoxon rank sum test.

### Phase II study

In the Phase II study, using a similar approach to Phase I, we collected a more limited dataset of practice list size, population density, and travel times to nearest specialist led hospital and Health Board headquarters for all non-urban general practices in Scotland (those located in SHS settlement categories 3–8), a total of 366 practices. These data were not circulated to the practices but were used for analysis in an identical approach to that in Phase I. In order to check that practices maintained similar relative positions in the hierarchy of peripherality in both studies, we examined the correlation between relative clinical peripherality index scores for practices included in both studies.

The study did not require formal ethical approval and received appropriate management approval from the Health Board responsible for services in these areas.

## Results

### Phase I Study

Characteristics of the practices are summarised in Table 3. Practices in the rural and remote LHCCs were generally characterised by low population density, with some variation attributable to population clusters in rural and remote small towns, smaller list sizes and longer travel times to secondary care and administrative centres, compared to urban practices.

**Table 3:** Practice characteristics by LHCC

LHCC	Ward population density (persons/hectare)	Practice list size (patients per practice)	Travel time to Secondary Care (minutes)	Travel time to NHS Board (minutes)
Argyll and Bute	0.6 (0.02,31.91)	870 (120,10013)	180 (53,375)	85 (1,368)
Lochaber	0.03 (0.02,16.85)	1199 (146,5130)	138 (53,243)	47 (2,148)
W. Ross, S. Skye and Lochalsh	0.02 (0.01,0.09)	1001 (223,2562)	117 (81,181)	117 (81,181)
Inverness	6.58 (3.78,32.21)	4359 (2231,9894)	4 (2,8)	4 (2,8)

Data are shown as median (inter-quartile range).

#### Factor analysis

The principal component method of factor extraction on the four candidate variables yielded a robust one-factor solution explaining 75% variance (Table 4). Factor loadings ranged from 0.83 to 0.89 (Table 5). The maximum likelihood method was also applied and yielded a similar one factor solution (data not shown). Since only one factor was extracted, no rotation took place.

**Table 4:** Eigen values and percent of variance derived from factor analysis *

Factor	Eigenvalue	Difference	Proportion of variance	Cumulative proportion
1	2.99	2.53	0.75	0.75
2	0.46	0.14	0.12	0.87
3	0.33	0.11	0.08	0.95
4	0.21	-	0.05	1.00

* Number of observations = 71;

Method: principal-component factors; One factor was retained, unrotated.

LR test: independent vs. saturated: chi^2 ^= 160.48. (p = 0.0001)

**Table 5:** Factor loadings identified using principal component method

Variable	Factor Loading	Uniqueness
Practice List Size	0.86	0.26
Practice Population Density	0.88	0.22
Travel time to nearest hospital	-0.89	0.21
Travel time to Health Board	-0.83	0.31

**Figure 2:**
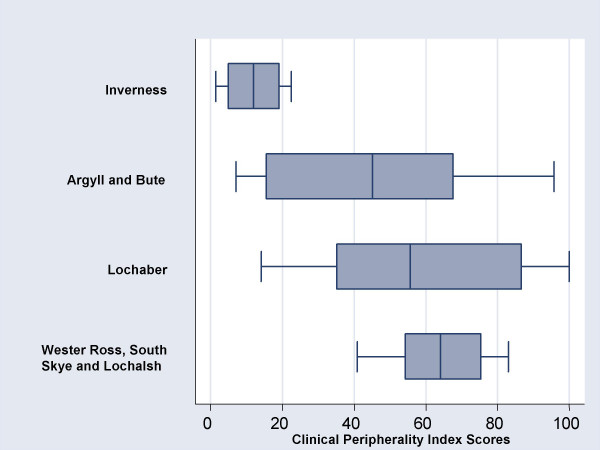
Clinical Peripherality Index scores for each LHCC.

The 3 rural and remote LHCCs had higher median values and a greater scatter of clinical peripherality index scores among their practices, compared to Inverness, the urban LHCC (Figure 2).

There was evidence that the pattern of services offered in practices and their mode of delivery was associated with the clinical peripherality index. Table 6 describes the median peripherality index associated with clinical and access characteristics, pattern of services offered and availability of professional support services in practices. Higher indices (greater peripherality) were associated with providing pharmacy services, cover for airports and mountain rescue services. More peripheral practices were less likely to have a practice nurse or administrator, and were less likely to be involved in teaching undergraduates or postgraduate students or to have a GP registrar (doctor in training) in the practice. The likelihood of having any access to a range of other professionals, health care assistants, counsellors, health visitors, district nurses, physiotherapists, community psychiatric nurses or chiropodists was not associated with peripherality, although the degree of access was often lower in more remote practices, for example infrequent visits by allied health professionals such as nurses or chiropodists.

**Table 6:** Clinical Peripherality Scores associated with characteristics of practices

	Service NOT provided			Service provided			
	n	Median	IQR	n	Median	IQR	p-value

**Clinical**							
Minor accident	12	64.8	30.3, 76.1	37	59.2	40.8, 78.9	0.6419
Single handed	33	50.7	31, 67.6	16	78.9	64.1, 90.8	0.0011
Pharmacy	15	29.6	23.9, 47.9	34	69.7	52.1, 84.5	0.0001
**Access**							
Island Location	29	56.3	32.4, 76.1	20	61.3	49.3, 87.3	0.1729
Hospital Cover	47	45.1	18.3, 67.6	24	66.9	30.3, 86.6	0.04
**Roles**							
GP Registrar	40	65.5	43, 81.7	9	32.4	15.5, 56.3	0.0062
UG Teaching	32	65.5	45.1, 83.8	17	43.7	31, 69	0.026
PG Training	42	64.1	43.7, 80.3	7	31	8.5, 33.8	0.0022
Occ Health	38	59.9	39.4, 76.1	11	71.8	32.4, 91.5	0.5981
Police Surgeon	33	60.6	39.4, 74.6	16	62	29.6, 90.8	0.5224
Airport Duties	43	56.3	33.8, 73.2	6	90.8	85.9, 93	0.0009
Rescue service	38	54.2	33.8, 73.2	11	78.9	57.7, 94.4	0.0259
Sports Medicine	43	60.6	35.2, 77.5	6	64.8	50.7, 93	0.5022
**Professional Support**							
Practice Nurse	13	74.6	64.8, 80.3	36	48.6	31.7, 71.1	0.0081
Practice HCA	44	62.7	40.1, 78.9	5	33.8	32.4, 43.7	0.1654
Administrator	13	83.1	62, 91.5	36	54.2	35.9, 71.1	0.0209
Counselling	43	60.6	36.6, 80.3	6	56.3	26.8, 70.4	0.4278
Health Visitor	2	62.7	31, 94.4	47	60.6	36.6, 77.5	0.7618
District Nurse	4	62	28.9, 94.4	45	60.6	39.4, 76.1	0.7424
Physiotherapy	12	70.4	45.8, 86.6	37	59.2	33.8, 73.2	0.1561
CPN	17	73.2	39.4, 88.7	32	59.9	35.9, 71.1	0.1928
Chiropodist	13	49.3	26.8, 80.3	36	61.3	41.5, 76.1	0.4687

### Phase II study

**Figure 3:**
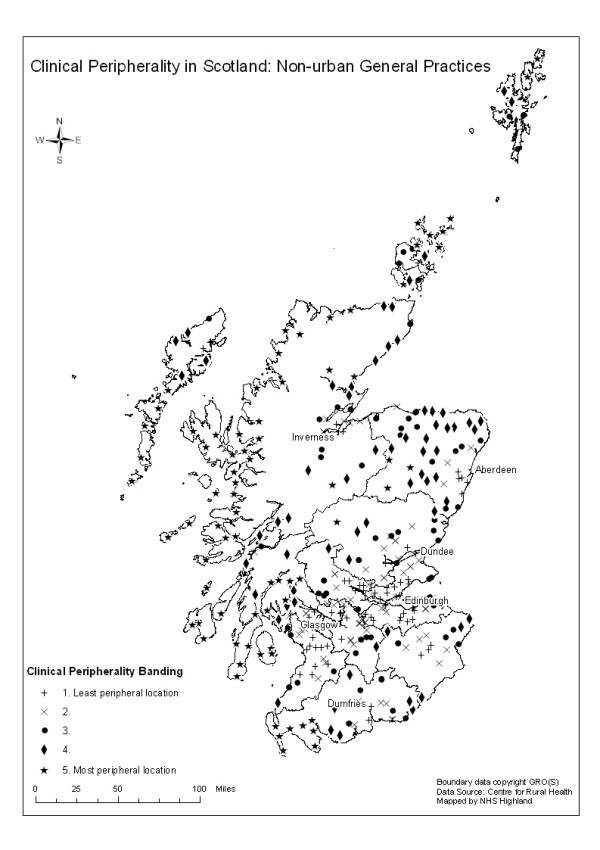
Clinical Peripherality banding for all non-urban practices in Scotland, expressed in quintiles.

A map showing the clinical peripherality indices for all non-urban practices in Scotland, expressed as quintiles, is shown in Figure 3. The most peripheral practices are generally located in the west of the country. Within the island groupings, such as Orkney, Shetland and Western Isles there is a spectrum of scores reflecting the proximity of some practices to the islands' hospital and Health Board headquarters which are located in the main small town of each island grouping, in contrast to the remoteness of some of the other islands within each archipelago. The relative clinical peripherality index scores for practices included in both analyses were highly correlated (Spearman's correlation coefficient = 0.8223, p = 0.001) indicating that these practices maintained similar relative positions in the hierarchy of peripherality.

## Discussion

In this exploratory study, we have derived a new index applied to rural and remote general practices in Scotland, which we have termed clinical peripherality. Underlying this model is the knowledge from previous literature that rural and remote practices are required to provide a broad spectrum of general medical services, often with a limited number of staff, to patients scattered over large geographic areas and often a long way from secondary medical care. The diverse nature of rural practice, where individual practitioners deliver patient care by taking on roles beyond the professional norm, has been previously reported in Scottish [4], UK [13] European [14], and Australian [15] literature. In this respect, rural practice differs substantially from its urban counterpart. Historically, many rural and remote areas have considered their individual local circumstances to be unique, but whilst there are local contextual factors in each area, there are nevertheless shared characteristics of rural and remote settings. The clinical peripherality index has potential value in conceptualising and modelling aspects of service provision and training needs for practitioners and has potential advantages over existing indices which merit testing in future studies. Although it bears some similarity to the Scottish Household Survey Classification of settlements, its focus on the characteristics of the general practice, namely its dependence on practice size, population density and the location of the nearest specialist facilities, gives it a specific relevance for health planners.

Two examples may illustrate future potential usage. In the Shetland, Orkney and Western Isles which are offshore island communities of Scotland, the Scottish Household Survey 8-fold Classification of Settlements would characterise the whole island as "very remote rural" and the main town of each island grouping as a "very remote small town". However, as each island grouping currently has a consultant led hospital providing acute medical and surgical services and Health Board headquarters located in the town, the peripherality scores for practices in or close to the town are low, implying that the nature of the practice may be more akin to an urban practice. However, if the island hospitals were to be downgraded such that local specialist services were no longer available, this would have a large impact on peripherality indices for these practices. Geographical access to specialist services would change, as has been shown in modelling studies by others [1]. This in turn could result in an alteration in services offered by the practices to a more peripheral model, especially if patients, as demonstrated elsewhere [2], chose to use local generalist services in preference to more distant specialists. Similar considerations in relation to education, training and engagement in management could apply if Health Boards were to be centralised. The clinical peripherality index offers planners an additional tool to model the effects of reconfiguration.

A second potential use for the index is to explore the relationship between peripherality, patterns of disease and quality of services offered by practices. NHS Scotland has recently introduced a Quality and Outcomes Framework (QOF) which requires practices to provide detailed reports to government on the prevalence of key indicator conditions and the percentage of patients receiving specific targeted treatments for these conditions in their practice. This in turn determines levels of payment to the practices. Since these data are collected at individual practice level, there is an opportunity to explore the relationship between peripherality, patterns of disease and quality of services offered by linking QOF data to the peripherality index for each practice. This offers the opportunity to determine whether more peripheral practices are systematically advantaged or disadvantaged.

Our study has some strengths and limitations. The Phase I study collected detailed information about three rural and remote LHCCs in Western Scotland. Multiple data sources were used to create practice community profiles, there was a high response rate in the Phase I study to circulation of the draft profiles (83%) and only 18 corrections (representing 0.39% of all data fields) were required among those who returned the profiles, therefore we are confident that the data are robust. Since the travel times were calculated from unit postcodes, there will be some underestimation where a practice and a hospital share a post code. This is more likely to apply in the urban setting, but the magnitude of difference between urban and rural settings suggests that this effect will be small. Because of the large number of practices in the Phase II study, we collected more limited data and did not circulate the profiles to individual practices. However, since only very few corrections were required by respondents to the Phase I study, and since only 3 of these were related to the candidate variables used in the Phase II study, we believe that the Phase II data are robust. The high correlation between relative clinical peripherality index scores for practices included in both Phases also indicates that practices maintained similar relative positions in the hierarchy of peripherality. Since the data were collected for this study, LHCCs have been phased out and replaced with a new organisational management structure of Community Health Partnerships, which cover larger geographical areas. However, there has been no reorganisation at individual practice level, and because the data were collected at the level of individual practices, the conclusions of the study remain valid.

The measure of peripherality we have chosen has some features of a gravity model, in which the economic influence of a centre on a peripheral location is proportional to the volume of activity at the centre (i.e. its "size") and inversely proportional to its distance from the peripheral location, hence the analogy with gravity. Our model differs in that we have assumed that in each case the influence of the centre (i.e. the specialist led hospital or Health Board) is constant, in that it provides the secondary services required. However, some features of the peripheral location itself will influence its peripherality, as these will impact on its requirement for interaction with the main centre. These might be thought of as contingent or associated factors of peripheral disadvantage. In any individual general practice, the number of patients served and the area over which they are dispersed influences the material and human resources available, their pattern of use within the practice and their interactions with the centre. This is exemplified by the wider range of services offered by the practitioners in these practices, i.e. the "generalist" approach. Distance to definitive hospital care is important for both acute emergencies and for complex disease management, while distance to decision-making and educational facilities has implications for personal development of practitioners. The increased distance to Health Board headquarters in more peripheral areas may lead to a weaker influence on governance, one of the contingent elements described by Copus [7]. Thus, we believe that the candidate variables chosen in this study are appropriate. Furthermore, both in the Phase I study, and in the subsequent, much larger study, a similar one factor solution was produced. The interpretations of the relationships among the variables do not change because of similar factor loadings in both the analyses. This suggests that the factor pattern is stable and that the solution can be re-produced in different samples and is thus robust.

Previous studies and policy documents have used varying measures of rurality to develop manpower and funding strategies for rural environments. [16,17]. Others have described rurality as a contributor to the difficulties of service provision, for example in maternity services [18] and in provision of nursing services [19]. It has been noted that groups are better represented when their rurality is determined by multi-factor descriptions [20,21] and the notion of describing rurality through 'bundles' of indicators is not new [13,17,22]. However, there has been no consensus on an appropriate measure of rurality for health service purposes and the most common explanation of note is based on a measurement of population density [23]. We suggest that clinical peripherality has advantages over previously used measures for the planning of health policy, both in relation to service provision and to training of staff for rural and remote locations.

There was evidence in this study that the range of services provided by individual practitioners was associated with clinical peripherality and further investigation of that relationship is required. Future work should also focus on aspatial peripherality [5,7], i.e. features not simply determined by geographic location such as IT infrastructure, social capital, institutional networks and others. In the future delivery of rural and remote health services, some of these aspatial concepts may become increasingly important, for example the use of telemedicine or increasingly sophisticated point of care testing.

## Conclusion

The clinical peripherality index combines features of gravity model-based and travel time/accessibility indicators. It has the potential to be applied to training of staff for rural and remote locations and to other aspects of health policy and planning.

## Competing interests

The author(s) declare that they have no competing interests.

## Authors' contributions

GS and DG conceived the study. GS undertook the data collection and analysis. SS undertook the statistical analyses. All authors contributed to writing the paper, and have read and approved the final manuscript.

## Pre-publication history

The pre-publication history for this paper can be accessed here:


